# Suicidal Ideation and Suicidal Attempts in Referred Adolescents with High Functioning Autism Spectrum Disorder and Comorbid Bipolar Disorder: A Pilot Study

**DOI:** 10.3390/brainsci10100750

**Published:** 2020-10-17

**Authors:** Gabriele Masi, Silvia Scullin, Antonio Narzisi, Pietro Muratori, Marinella Paciello, Deborah Fabiani, Francesca Lenzi, Maria Mucci, Giulia D’Acunto

**Affiliations:** 1 IRCCS Stella Maris, Scientific Institute of Child Neurology and Psychiatry, Calambrone, 56018 Pisa, Italy; silviascullin@gmail.com (S.S.); antonio.narzisi@fsm.unipi.it (A.N.); pmuratori@fsm.unipi.it (P.M.); deborah.fabiani@gmail.com (D.F.); flenzi@fsm.unipi.it (F.L.); mmucci@fsm.unipi.it (M.M.); gdacunto@fsm.unipi.it (G.D.); 2 Faculty of Psychology, Università Telematica Internazionale Uninettuno, 00186 Rome, Italy; m.paciello@uninettunouniversity.net

**Keywords:** autism spectrum disorder, bipolar disorder, suicidal ideation, suicidal attempts, adolescence

## Abstract

Suicidal ideation and attempts in adolescents are closely associated to bipolar disorders (BD). Growing evidence also suggests that high functioning autism spectrum disorders (HF-ASD) are at increased risk for suicidal ideation and behaviors. Although BD and HF-ASD are frequently comorbid, no studies explored suicidality in these individuals. This exploratory study addressed this issue in a clinical group of inpatient adolescents referred to a psychiatric emergency unit. Seventeen adolescents with BD and HF-ASD and severe suicidal ideation or attempts (BD-ASD-S), were compared to 17 adolescents with BD and HF-ASD without suicidal ideation or attempts (BD-ASD-noS), and to 18 adolescents with BD and suicidal ideation or attempts without ASD (BD-noASD-S), using a structured assessment methodology. Individuals with BD-ASD-S had a higher intelligence quotient, more severe clinical impairment, more lethality in suicide attempts, more internalizing symptoms, less impulsiveness, and lower social competence. Severity of ASD traits in individuals and parents did not correlate with suicidal risk. Some dimensions of resilience were protective in terms of repulsion by life and attraction to death. Main limitations are the small sample size, the lack of a control group of typically developing adolescents. However, a better understanding of the specificities of bipolar HF-ASD individuals with suicidality may improve prevention and treatment strategies.

## 1. Introduction

The concept of suicidality includes both suicidal ideation, with or without a plan, and with a wide range of severity, and suicidal attempts, that is self-injurious behaviors intended to kill oneself, with or without medical implications, but nonfatal, while completed suicides are fatal [1]. The term suicidality has long fallen out of favor with psychiatrists and psychologists, due to its imprecision, as it includes heterogeneous clinical conditions, not only in terms of presentation, but also in etiology, diagnosis, prognosis, and treatment. Disentangling this wide range of individuals in more specific subgroups, according to age and gender, psychiatric diagnoses, neurodevelopmental pathways, personality traits, psychiatric familial load, previous traumatic experiences, and social difficulties, may favor a more precise definition of risk factors, and more focused and appropriate prevention and treatment strategies [2,3].

There is growing evidence that a diagnosis of Autism Spectrum Disorders (ASD) is one of the risk factors for suicide [4,5]. In a population-based cohort of ASD probands (*n* = 27,122, all diagnosed between 1987 and 2009) compared with gender-, age-, and county of residence-matched controls (*n* = 2,672,185), Hirvikoski et al. [6] reported a probability for ASD to die by suicide 10 times greater than in the general population. In a clinical cohort study, including 367 high functioning individuals with ASD, 243 (66%) presented self-reported suicidal ideation, and 127 (35%) self-reported plans or attempts at suicide. They more likely reported lifetime experience of suicidal ideation than the UK population sample (odds ratio 9.6 (95% CI 7.6–11.9), *p* < 0.0001), than people with one, two, or more medical illnesses (*p* < 0.0001), or with psychotic illness (*p* = 0.019) [7].

Suicidality in high functioning (HF)-ASD can present with specific features [4]. Thoughts can become easily ruminative, not only in terms of triggering situations for suicidal ideation (i.e., impaired interpretation of social relationships), but also in methods of searching information aimed at planning a suicide attempt [8]. Furthermore, the high lethality and violence of the suicidal methods in ASD individuals (such as hanging, gunshot, railway hitting, or jumping off a bridge) increases the risk of a completed suicide during an attempt, compared to other psychiatric populations [9]. This evidence suggests that ASD should be considered a specific and independent risk factor for suicide [10,11].

Studies specifically addressing suicidal risk factors in youth with ASD have underlined the role of extreme loneliness [12,13], social adverse experiences [14,15], previous traumas [16], internalizing symptoms (anxiety/mood disorders), low emotional regulation, and ADHD [16,17], particularly in HF-ASD individuals [18,19,20]. Some cognitive features, such as decreased cognitive flexibility, insight of disease, and communication skills may flatten the expression of subjective suffering [21,22]. This is particularly true in adolescence, the age range with the highest suicidal risk [4]. HF-ASD adolescents are mostly exposed to increased social pressure, they can become more acutely aware of their disability, and they have to face the unbridgeable gap between themselves and neurotypical peers [22].

Among the factor increasing suicide risk, mood disorders, have been shown to be strongly related to suicidal outcomes [23]. Even if depressive disorders have been traditionally linked to the concept of suicidality [24], bipolar disorder (BD) has increasingly become, in the last decades, even more closely related to suicidal risk [25]. A systematic review (1970–2017) including articles on completed suicide in individuals with BD showed that suicide rates in BD are approximately 20- to 30-fold greater than in general population, with higher risk in BD-II individuals, and with a heritability of completed suicide of about 40%. Factors related to completed suicide are early onset, family history of suicide among first-degree relatives, previous attempted suicides, type of treatment, somatic, and psychiatric comorbidities [25].

Psychiatric comorbidity in ASD is still under-investigated, even if nearly 70% of people with ASD, including children and adolescents, experience at least one comorbid psychiatric disorder, and around 40% may have two or more psychiatric disorders [9,26,27]. Regarding the different comorbidities in ASD, the prevalence of mood disorder among 21,797 participants with ASD has been reported as 18.8% (95% CI: 10.6–31.1) [28]. The prevalence of depressive disorders has been reported by Lai and colleagues as 11% (95% CI: 9–13) in children and adults [29], and by Hudson and colleagues (meta-analysis including 66 studies) as 12.3% (95% CI: 9.7–15.5) and 14.4% (95% CI: 10.3–19.8) (current and lifetime prevalence, respectively) [30].

The BD-ASD association has been more recently highlighted, along with its important clinical and therapeutic implications [31]. Two reviews assessed the co-occurrence of bipolar disorders in ASD [29,32]. Vannucchi and colleagues found that the prevalence of bipolar disorders ranged from 6% to 21.4% across studies [32], whereas Lai and colleagues reported a prevalence of 5% (95% CI: 3–6) among 153,192 ASD individuals [29]. Regarding only children and adolescents, Joshi and colleagues [31] found that 30% (47/155) of bipolar I probands met criteria for ASD (diagnosis based on DSM-III-R criteria).

While both BD and HF-ASD represent independent risk factors for suicidal ideation and behavior, their association in terms of increased suicidal risk is strongly under-explored in clinical samples. Dell’Osso and colleagues [33] investigated the prevalence of suicidal ideation in 34 adult individuals with ASD without intellectual disability, 68 with subthreshold ASD (“autistic traits”), and 160 healthy controls. Individuals with ASD reported significantly higher scores than the other two groups in mood disorders and depression, while the subthreshold autistic individuals presented higher scores than the heathy control group. Of note, both individuals with ASD and subthreshold ASD scored higher in suicidality, compared to healthy controls, without significant differences between clinical and subthreshold ASD individuals. The depressive score and the restricted interests and rumination domain score were the strongest predictors of suicidality. These results underline the association between mood spectrum and suicidality in adult individuals with ASD, including those with subthreshold forms. These correlations have not been explored in adolescents with ASD, albeit the high rates of suicidal ideation and attempts in this age range.

Among the different personality features possibly related to suicidal risk in adolescents, three domains were considered as particularly significant, according to the available literature, the attitude to life and death, the impulsivity, and the resilience to life stressors. The repulsion by life and the attraction (or low fearlessness) about death have been proved to be related to suicidality in adolescents with psychiatric diagnoses [34,35]. Highly impulsive adolescents tend to act rashly in the context of negative emotions, because long-term benefits are less important than the immediate short-term gains of emotion regulation [36]. Finally, life adversities or stressful life events increase the risk of suicidal ideation and behaviors) [3], and low adaptive resilience abilities after stressful life events are a further vulnerability factor and a possible target for interventions [37].

The aim of our study is to explore if within the bipolar spectrum, adolescents with ASD and suicidality may present specificities in three personality dimensions usually related to suicidality in adolescence (resilience, impulsivity, and attitude to life and death). We compared subjects with BD, ASD, and suicidality (Group 1), with BD; and suicidality, but without ASD (Group 2); and BD and ASD, but without suicidality (Group 3), in order to possibly disentangle the role of autism and suicidality in bipolarity. We hypothesized that bipolar adolescents with HF-ASD could present specific clinical characteristics in their suicidal manifestations, including clinical severity, cognitive abilities, psychiatric comorbidities, and personality features, compared to those without suicidality or without ASD, which may represent possible targets for the diagnostic procedures and treatment plans. Given the exploratory design of the study, we investigated these psychological features without a-priori hypotheses.

## 2. Materials and Methods

### 2.1. Sample

This was a naturalistic study based on a clinical database of 52 adolescents (age range 11 to 18 years) with BD, all referred as individuals to our psychiatric emergency unit between January 2018 and July 2019. Seventeen individuals (age range 11 to 18 years) had a comorbid HF-ASD and severe suicidal ideation or suicide attempt(s) (BD-ASD-S group), 17 (age range 11 to 18 years) a comorbid HF-ASD, but without suicidal ideation or suicidal attempt(s) (BD-ASD-noS group), and 18 (age range 11 to 18 years) with severe suicidal ideation or suicide attempts, but not ASD (BD-NoASD-S). The diagnosis of BD was based on DSM 5 criteria, and a diagnostic interview, the Kiddie Schedule for Affective Disorders and Schizophrenia for School-Aged Children-Present and Lifetime Version (K-SADS-PL) [38], administered to the patient and at least one parent.

The diagnosis of ASD was based on the DSM 5 diagnostic criteria [39], and confirmed with the module 3 of Autism Diagnostic Observation Schedule—Second Version (ADOS-2) [40]. Only a minority of individuals’ caregivers were interviewed with the Autism Diagnostic Interview-Revised (ADI-R) [41]. The ADI-R is less effective in the diagnosis of HF-ASDA and it may not recognize more subtle forms of ASD [42]. It is therefore possible that, in these individuals with HF-ASD, the symptoms may not be evident at 4 to 5 years of age (developmental period investigated in the ADI-R), but could emerge only when social demands exceed these individuals’ limited capacities [39].

The diagnosis of severe suicidal ideation or suicide attempt was based on a score 3 or above at the Columbia–Suicide Severity Rating Scale [CSSRS] [43]. All the individuals presented normal intelligence (Full scale IQ above 85), based on the Wechsler Intelligence Scale for Children—Fourth Edition (WISC-IV) [44].

### 2.2. Measures

All the individuals received a diagnostic assessment with the K-SADS-PL, a semi-structured interview to diagnose childhood mental disorders in children aged 6 to 18, administered by trained child psychiatrists, in order to diagnose BD (including Type I, Type II, and type NOS), ASD, and comorbidities. All the individuals were assessed with the WISC-IV, to exclude an Intellectual Disability.

The ADOS-2 was administered to support the diagnosis of ASD to all the individuals with first diagnosis of ASD based on historical information and the diagnostic interview K-SADS-PL. The ADOS-2 is a semi-structured interaction that measures symptoms of autism through a standard set of probes. It provides an empirically derived algorithm that differentiates children with ASDs from those with other delays or with typical development. The ADOS-Calibrated Severity Score was used to assess the severity of autistic symptoms [45].

The global clinical severity was assessed with the Clinical Global Impression Severity (CGI-S) [46], while the functional impairment with the Child Global Assessment Scale (C-GAS) [47].

For a dimensional assessment of psychopathology, all individuals were assessed with the Child Behavior Checklist (CBCL) [42,48], a 118-item scale, completed by parents, assessing how often a certain behavior applies to their offspring, on a three-point scale (0 = absent, 1 = occurs sometimes, 2 = occurs often), clustered in two broad-band scores, designated as Internalizing Problems and Externalizing Problems, a Total Problem Score, and with 8 different syndromes scales (Withdrawal, Somatic complaints, Anxiety/depression, Social problems, Thought problems, Attention, Rule-breaking behavior, Aggressive behavior). In the current study, we assessed the presence of Emotional Dysregulation using the CBCL Dysregulation Profile (CBCL-DP), based on the sum of t-scores of the three CBCL subscales, Anxious/depression, Attention problems, and Aggressive behaviors [43,49]. The reliability coefficients (Cronbach) of CBCL Attention Problems, Aggression, and Anxious/Depressed subscales were 0.82, 0.81, and 0.82, respectively.

Type of suicidality and severity of suicidal ideation and behavior were assessed using the Columbia–Suicide Severity Rating Scale (CSSRS), (score 3 or higher), recommended by the Center for Disease Control and Prevention and Food and Drug Administration for the assessment of adolescents at high suicidal risk.

In order to explore further psychological features possibly related to suicidal risk, that is attitude to life and death, impulsivity, and resilience abilities facing life adversities, three specific measures were included in the assessment for all the individuals included in the study.

The Multi-Attitude Suicide Tendency Scale (MAST) [50] was used to assess attitude for life and death, related to the fearlessness about death, and to the capability for suicide. This measure, designed to assess suicidal tendencies in youth, is a 30-item scale exploring four types of attitudes: attraction to life, repulsion by life, attraction to death, and repulsion by death. All four factor scales showed good reliability estimates, as well as relationships with measures of suicidal behavior and ideation and general psychopathology [50,51].

Impulsivity was assessed with the Barratt Impulsiveness Scale-11 (BIS-11) [52,53], including 30 items that are scored to yield second-order factors, Attentional, Motor, and Non-planning impulsiveness.

Resilience was explored with the Resilience scale for Adolescent (READ) [54,55,56], a self-administered 28-item questionnaire, with a score for each item ranging from 1 (totally disagree) to 5 (totally agree), which incorporates intrapersonal and interpersonal protective factors mapping onto the three salient domains of resilience, including individual, family, and external environment. Confirmatory factor analysis validated the original five-factor structure of the READ, including Personal Competence, Social Competence, Structured Style, Family Cohesion, and Social Resources. The measures showed good reliability and validity in adolescents [57].

All participants and parents were informed about the assessment instruments. Written informed consent was obtained from participants and parents. The study conformed to Declaration of Helsinki; the Ethics Committee of the Hospital approved the methodology of the study (Identification Code 2014/0001507).

### 2.3. Statistical Analyses

Descriptive analyses were used to describe demographic and clinical characteristics of the whole sample. Chi-square analyses were performed on categorical variables, and a t-test or one-way ANOVA on continuous variables, with statistical significance set at 0.05. The Bonferroni–Holm method was used for multiple comparisons. The Student’s t-test, with Bonferroni correction, was used in order to compare the calibrated severity score of ADOS-2 between BD-ASD-S and BD-ASD-noS.

A structural equation modeling (SEM) was conducted to examine the association between resilience protective factors measured by READ scales and suicide tendency measured by the MAST (attraction and/or repulsion to life and/or death) in all individuals. The SEM model has been tested using the maximum likelihood (ML) method since none of the variables exceed the value of |1| for univariate skewness and kurtosis. To examine model fit, several goodness-of-fit indices were used: the comparative fit index (CFI), the Tucker and Lewis index (TLI), the standardized root mean square residual (SRMR), and the root mean square error of approximation (RMSEA).

## 3. Results

The three groups BD-ASD-S, BD-noASD—S, and BD-ASD-noS did not differ according to mean age (14.53 ± 2.03 years, 14.78 ± 1.86 years and 14.94 ± 2.22 years, respectively, F = 0.175, df = 51, *p* = 0.840), while gender ratio was uneven, as in the BD-ASD-S the male/female was 14/3, in BD-noASD-S 6/12, and in BD-ASD-noS 10/7, (χ^2^ = 8.62, df = 2, *p* = 0.013). Regarding the frequency of the BD types (BD I, BD II, and BD Not Otherwise Specified) in the three groups, they were respectively, six, seven, and five in the BD-ASD-S; six, eight, and three in the BD-noASD—S; and seven, five, and five in the BD-ASD-noS. Differences among groups were not statistically significant (χ^2^ = 0.92, df = 4, *p* = 0.921, ns).

The intellectual quotient, measured with the WISC-IV, was significantly higher in the BD-ASD-S group than in the other two groups for the full scale IQ and for the verbal comprehension index, while for the perceptual reasoning and working memory indices, BD-ASD-S scored significantly higher than BD-ASD-NoS. The processing speed index did not differ among groups (Table 1).

Regarding clinical severity, assessed with the CGI-S, the BD-ASD-S group was the most severely impaired (6.41 ± 0.71), compared to the BD-noASD-S (6.00 ± 0.68), and BD-ASD-noS (4.82 ± 0.95) (F = 18.5, *p* < 0.001). Similarly, the BD-ASD-S group presented the greatest functional impairment, assessed with the C-GAS (28.76 ± 7.35), compared to the BD-noASD-S (35.06 ± 7.11), and the BD-ASD-noS (41.47 ± 7.97) (F = 12.25, *p* < 0.001).

According to the type of suicidality, assessed with the CSSI-R, types of suicidal ideation, frequency of ideation, control over ideation, deterrents from suicide behavior, and reasons for ideation did not differ among groups. Preparatory acts did not differ, but potential lethality higher than 1 was more frequent in the BD-ASD-S group (41.2%), compared to the BD-noASD-S group (11.1%) (*p* = 0.042).

Regarding psychiatric diagnoses, according the K-SADS-PL, only obsessive compulsive disorder was more frequently reported in individuals with BD-ASD-S (70.6%), and with BD-ASD-noS (41.2%), compared to those with BD-noASD-S (5.6%) (χ^2^ = 15.70, df = 2, *p* < 0.001 for both BD- ASD-S and BD-ASD-noS after Bonferroni–Helm method). All the other categorical diagnoses (ADHD, anxiety disorders, depression, and disruptive behavior disorders) did not differ among groups.

Regarding the comparison among groups according to the CBCL, individuals with BD-ASD-S presented higher scores in the internalizing problems, and, among the syndrome scales, in the thought disorders scale, while the anxiety-depressed and the withdrawal scales only approached statistical significance, and the three groups did not differ according to the dysregulation profile score (Table 2).

Autism severity according to ADOS-Calibrated Severity Score, did not differ significantly between suicidal and non-suicidal individuals with ASD (BD-ASD-S: M = 5.85, SD = 1.15 versus BD-ASD-noS: M = 5.59, SD = 0.76 t = 0.701, *p* = 0.243).

All the four scales of the prevalent attitude (repulsion or attraction) for life and death (attraction to life, repulsion by life, attraction to death, and repulsion by death) failed to reach statistical difference among groups.

Similarly, the attentional and motor impulsivity, as well as the total score of the BIS, did not differ in the three groups. On the contrary, the non-planned impulsivity was higher in the BD-noASD-S group, compared to BD-ASD-S and BD-ASD-noS groups (F = 3.85, *p* = 0.028). More specifically, in multiple comparisons, ASD suicidal individuals presented less non planned impulsivity than individuals without ASD (*p* = 0.040).

On the Resilience test (READ), only Personal Competence differed among groups (F = 6.85, *p* = 0.004), as, after multiple comparisons, individuals with BD-ASD-noS significantly outscored those with BD-noASD-S (*p* = 0.003). All the other four dimensions of the scale (Social Competence, Structured Style, Family Cohesion, and Social Resources), as well as total score, were similar among groups.

**Figure 1:**
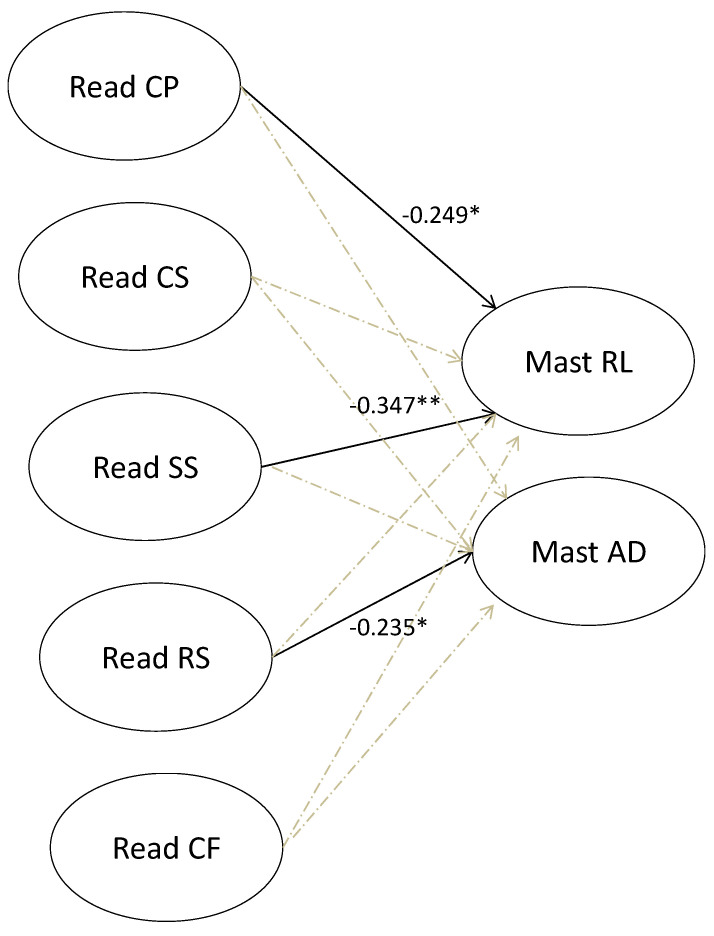
Relationships between Resiliency and Suicide Tendency. Note: READ CP = Personal Competence, READ CS = Social Competence, READ SS = Structured Style, READ RS = Social Resources, READ CF = Family Cohesion, Mast RL = repulsion by life, Mast AD = attraction to death. *: *p* < 0.05, **: *p* < 0.01.

Results of the path analysis (χ^2^ (7) = 6.232, *p* = ns, CFI = 1, TLI = 1; SRMR = 0.057, RMSEA = 0.000 (0.000–0.164), *p* = ns) attest that some resilience dimensions were significantly associated with suicide tendency. In particular, personal competences (unstandardized coefficient: −0.19, S.E. = 0.09) and structured style (unstandardized coefficient: −0.29, S.E. = 0.10) were negatively associated with repulsion by life, and social resource (unstandardized coefficient: −0.24, S.E. = 0.12) was negatively associated with attraction to death. The model explained the 25% of repulsion by life variance, and 0.05% of attraction to death variance (Figure 1).

## 4. Discussion

If BD is a well-known risk factor for suicide ideation and behavior, this risk may be markedly increased in bipolar individuals with HF-ASD. In fact, persons with HF-ASD experience the world in a specific way, compared with typically developing individuals, from basic information processing and attribution processing patterns, to social perspectives, such as the difficulties in expressing feelings and communicating with others [4,8,32,53,54,58]. A better comprehension of possible peculiarities in a very special population with both BD and HF-ASD may improve our understanding on how suicidal behaviors may be recognized, managed and treated. In our study, suicidal bipolar individuals with ASD were more frequently males, and presented greater clinical severity and functional impairment. Furthermore, they presented higher potential lethality of their suicidality, compared to the group without ASD, consistently with literature findings [17,32,55,56,59,60]. These differences were not accounted for by the type of BD, which did not differ among groups.

The suicidal individuals with ASD presented also a higher IQ, suggesting that the psychopathological severity has a greater impact on global functioning than their cognitive functioning [57,61]. Consistently, studies on populations with neurodevelopmental disorders reported that individuals with intellectual disabilities show a lower suicide rate than general population [57,58,61,62]. A high IQ may represent a specific risk factor [59,60,63,64], as it may imply an increased self-consciousness of one’s own disability, leading to painful feelings of inadequacy, guilt, and exclusion from peer groups. Such aspects may be more evident during adolescence, when heightened social requests can easily go beyond the individual’s coping abilities, further worsening the social withdrawal [58,61,62,63,65,66,67,68].

Regarding psychiatric diagnoses, only OCD differed among groups, and was more frequently reported in ASD individuals, irrespective of the presence of suicidality. However, a recent study showed that rumination was significantly associated with a history of attempted suicide in a sample of 75 adults with ASD [8,16].

Suicidal individuals with BD-ASD individuals presented at the CBCL (completed by parents) significantly more internalizing problems and thought problems. Internalized symptoms can represent a risk factor for suicide in individuals with ASD [16,24]. Hence, investigating symptoms with standardized scales with both individuals and caregivers, including direct psychiatric interviews, is crucial, given that many individuals with HF-ASD are able to “camouflage” internalized symptoms.

The severity of autistic traits in individuals with or without suicidality, according to ADOS-2, did not differ between the two groups, suggesting that this feature does not affect the suicidal risk. Available evidence on this topic is inconsistent. While a number of studies on “Asperger syndrome” populations have shown a negative correlation between suicidal ideation and autistic traits [15,23,67,69], on the contrary Cassidy et al. [7,15] reported that persons diagnosed with Asperger syndrome with previous suicide attempts showed significantly higher autistic traits than individuals with this diagnosis without a history of attempted suicide.

In our study, the three groups did not differ according to repulsion or attraction for life and death. On the contrary, non-planned impulsivity was highest in the BD-noASD-S group, compared to both the ASD groups (with or without suicidality), suggesting that in individuals with ASD the suicidality may be less impulsive than in individuals without ASD, although both the groups have a diagnosis of BD This could imply a “colder” transition from ideation to suicide attempt, leading to more lethal strategies [68,70].

Regarding resilience, personal competence was lowest in individuals with BD-noASD-S individuals, and highest in those with BD-non suicidal ASD, with intermediate scores in the BD-ASD-S group, suggesting that the feeling of a low personal competence is more urgent in non-autistic individuals, and namely in those who present suicidal ideation or behavior, in whom it may have a triggering role in their suicidality.

A structural equation modeling was applied to examine the influences among variables that may interact, namely, between resilience protective factors, and suicide tendency, in terms of attraction and/or repulsion to life and/or death. Some dimensions of resilience, particularly personal competences and structured style were protective in terms of repulsion by life, while social resource was protective in terms of attraction to death. This information may guide possible therapeutic interventions aimed at reinforcing specific areas of resilience in order to improve some risk factors related to suicide vulnerability in adolescence.

Our study shows a number of limitations that could limit the generalization of the results. Major limitations of the study are the small sample size, and the lack of a control group of typically developing adolescents. Furthermore, these groups were not recruited through an age- and gender-matching protocol design, although no statistically significant age difference emerged, and the female component was only scarcely represented. Finally, only a selected number of features were considered as relevant, and the diagnostic exploration did not include other potentially important elements.

Our study provides a first, exploratory contribution for better understanding the peculiarities of adolescents with BD and ASD and severe suicide ideation or suicide attempts, helpful for subsequent targeted examination of suicidality in autism/bipolar disorder. These specificities may help clinicians not only in the diagnostic process, focusing investigations on specific areas, but also in the prevention and treatment strategies, for planning timely and finely customized educational and therapeutic approaches, at the individual level, contributing to a precision medicine even in these complex and often misunderstood individuals.

## Figures and Tables

**Table 1:** Wechsler Intelligence Scale for Children—Fourth Edition (WISC-IV) (Wechsler, 2003). Comparisons among groups. Legenda; BD-ASD-S = Bipolar Disorder + High Functioning Autism Spectrum Disorder + Severe Suicide Ideation or Attempt; BD-noASD-S = Bipolar Disorder + Severe Suicide Ideation or Attempt, without High Functioning Autism Spectrum Disorder; BD-ASD-noS = Bipolar Disorder + High Functioning Autism Spectrum Disorder, without Severe Suicide Ideation or Attempt; SD: Standard Deviations; FSIQ: Total Intellectual Quotient; VCI: Verbal Comprehension Index; PRI: Perceptual Reasoning Index; WMI: Working Memory Index; PSI: Processing Speed Index. Statistical significance at *p* < 0.05.

	Group 1	Group 2	Group 3	One-Way	Bonferroni–Holm		
	BD-ASD-S	BD-no ASD-S	BD-ASD-NoS	ANOVA	Gr1 vs. Gr.2	Gr.1 vs. Gr.3	Gr.2 vs. Gr.3
	Mean; SD	Mean; SD	Mean; SD	(F); *p*	*p*	*p*	*p*
FSIQ	112.9; 16.2	97.3; 13.2	84.8; 18.8	(9.43); 0.001	0.041	0.000	ns
VCI	116.6; 18.4	99.8; 15.1	91.0; 17.1	(7.67); 0.002	0.029	0.002	ns
PRI	115.5; 16.9	104.2; 13.2	96.9; 19.8	(4.12); 0.024	Ns	0.023	ns
WMI	102.4; 14.1	91.4; 11.7	80.0; 12.1	(7.59); 0.002	Ns	0.001	ns
PSI	92.3; 10.7	94.94; 18.4	80.0; 18.4	(2.43); ns	Ns	ns	ns

**Table 2:** Child Behavior Checklist (CBCL): Comparisons among groups. Legenda: BD-ASD-S = Bipolar Disorder + High Functioning Autism Spectrum Disorder + Severe Suicide Ideation or Attempt; BD-noASD-S = Bipolar Disorder + Severe Suicide Ideation or Attempt, without High Functioning Autism Spectrum Disorder; BD-ASD-noS = Bipolar Disorder + High Functioning Autism Spectrum Disorder, without Severe Suicide Ideation or Attempt. Statistical significance at *p* < 0.05.

	Group 1	Group 2	Group 3	One-Way			
	BD-ASD-S	BD-no ASD-S	BD-ASD-NoS	ANOVA	Gr1 vs. Gr.2	Gr.1 vs. Gr.3	Gr.2 vs. Gr.3
	Mean; SD	Mean; SD	Mean; SD	(F); *p*	*p*	*p*	*p*
Internalizing	72.7; 7.6	69.9; 4.8	66.4;9.1	(3.1); ns	ns	0.049	ns
Externalizing	60.8; 7.9	63.2; 9.1	60.7; 6.4	(0.57); ns	ns	ns	ns
Total	68.1; 6.2	67.6; 5.6	65.6; 6.7	(0.79); ns	ns	ns	ns
Anxious/Depressed	75.1; 9.8	72.9; 9.2	66.9; 10.8	(3.1); ns	ns	ns	ns
Withdrawn	75.7; 9.3	73.2; 11.3	67.3; 9,9	(3.0); ns	ns	ns	ns
Somatic Complaints	62.8; 9.9	59.3; 7.0	59.2; 9.3	(0.93); ns	ns	ns	ns
Social Problems	65.9; 8.2	64.9; 7.6	67.4; 7.4	(0.46); ns	ns	ns	ns
Thought Problems	70.9; 9.3	70.2; 5.3	63.28: 9.2	(4.7); 0.013	ns	0.023	0.042
Attention	59.4.; 4.9	62.9; 8.9	64.2; 7.9	(1.9); ns	ns	ns	ns
Rule Breaking Problems	59.5; 6.2	627; 8.8	58,8; 5.83	(1.5); ns	ns	ns	ns
Aggressive behavior	61.9; 9.4	64.8; 9.5	60.9; 6.1	(1.0); ns	ns	ns	ns
Dysregulation Profile	196.4; 16.0	200.7; 19.1	192.1; 19.7	(0.96); ns	ns	ns	ns
